# The possible roles of human *Alu* elements in aging

**DOI:** 10.3389/fgene.2013.00096

**Published:** 2013-05-28

**Authors:** O. E. Mustafina

**Affiliations:** Institute of Biochemistry and Genetics, Ufa Research Center, Russian Academy of SciencesUfa, Russia

The huge amount of knowledge about the organization and functions of genome structural units accumulated through genome research allows us to examine cell aging processes in detail and to test existing hypotheses of aging. One of the hypothesis currently of interest is that instability of the cell genome is one of the main causes of aging (Vijg and Suh, 2013). Putative causes of genome instability during aging include breakage of double-stranded DNA, telomere shortening, activation of mobile elements, and decreased efficiency of repair systems (Chen et al., 2007; Aubert and Lansdorp, 2008; Maxwell et al., 2011). It is suggested that genome instability of somatic cells has an impact on gene expression and results in disturbance of cell processes, cessation of cellular growth, degeneration and atrophy of cells and tissues as well as aging of the whole organism. The role of mobile elements, in particular *Alu* retrotransposons, in genome instability and aging deserves special attention. Important questions include whether *Alu* elements in the human genome are an endogenous source of DNA damage and genome instability and whether they can promote aging of an organism and make a significant contribution to lifespan variation. *Alu* elements are characterized by considerable polymorphism in human populations. We asked, therefore, whether polymorphism of *Alu* elements can influence the human lifespan.

Opinions about mobile genetic elements have changed radically over the last two decades. Originally, they were characterized as selfish DNA but today we recognize their role in the organization of a genome and the regulation of gene expression (Deininger, 2011). *Alu* elements are classified as short interspersed elements (SINEs). The human genome contains about ~10^6^ copies of *Alu* retrotransposons and they represent ~10.6% of nuclear DNA. The distributions of different *Alu* elements within a one chromosome and between different chromosomes are uneven but are not random. *Alu* elements in human chromosomes 14, 16, and 21 are concentrated in centromeric areas, but clusters of *Alu* elements are not found in chromosomes 4, 19, 20, X or Y. The distribution of *Alu* elements is correlated positively with the presence of GpC-containing genome sequences and the distribution of protein-coding genes. These repeating elements are clustered near the genes controlling metabolic, transport, and signaling processes (Grover et al., 2003).

Genome instability has been found for all sites containing *Alu* elements, which serve as “substrates” for homologous recombination owing to their high frequency of occurrence in the eukaryotic genome and the identity of their sequences. Deletions and duplications can appear as the result of crossing-over between similarly oriented elements; e.g., between inversions of opposite orientation (Kolomietz et al., 2002). The existence of an inserted *Alu* element (*Alu*Y) is a predictor of increased recombination variability within 2 kb of the *Alu* element (Witherspoon et al., 2009). As a result of the analysis of human DNA sequences adjoining the site of recombination, the 26 nt sequence of an *Alu* element was found within the site or at a distance of 20–50 bp from it. This sequence is similar to that of a χ site, which stimulates recombination in *Escherichia coli*. Further, a sequence with homology to a translysine-binding site was detected within the *Alu* element. This protein is involved in partial untwisting of the DNA helix and its linkage with DNA results in increased sensitivity to the action of nucleases and greater probability of recombination (Martinelli et al., 2000). Thus, a large number of *Alu* elements in the genome and the existence of protein-binding sites in sequences involved in recombination lead to their functioning as potential sites for recombination and, perhaps, promotion of this process.

*Alu* elements are 7SL RNA-like SINEs (Deininger, 2011). Owing to structural features and various functions, *Alu* elements can participate in the regulation of gene expression and likely influence the expression of many genes by insertion into or close by gene promoter regions. *Alu* elements contain binding sites for nuclear hormone receptor complexes and a large number of functionally active transcription factors (Polak and Domany, 2006; Deininger, 2011). These sites can compete for linkage of transcription factors with gene promoters or act as promoters for nearby genes. For example, ~90% of sites responsible for the linkage of retinoic acid are located in *Alu* elements (Laperriere et al., 2007). Human aging is characterized by dysregulation of alternative splicing (Harries et al., 2011) and *Alu* elements can interfere with the mechanism underlying gene splicing. The presence of *Alu* elements in non-translation sites of a gene can result in alternative or aberrant splice sites. About 5% of all human alternative exons contain *Alu* sequences (Sorek et al., 2002). One of the consequences of the insertion of *Alu* elements into protein-coding sequences is the occurrence of an additional stop codon and a premature stop of translation resulting in the development of different diseases (Hancks and Kazazian et al., 2012). For example, the insertion of an *Alu* element into intron 18 of the human factor VIII gene leads to the absence of exon 19 during the splicing process, which results in development of the severe form of hemophilia (Ganguly et al., 2003). *Alu* elements can act as anti-sense regulators of transcription. *Alu* elements in gene introns might be located in anti-sense orientation regarding the direction of gene transcription; therefore, anti-sense RNA complementary to mRNA can be synthesized, and this is able to suppress splicing and mRNA translation. Anti-sense interactions of *Alu* transcripts with mRNA likely have a major role in the regulation of translation, degradation of mRNA, and change of gene transcription (Häsler and Strub, 2006). The insertion of *Alu* elements into genes creates alternative sites of polyadenylation, which is one of the important stages of mRNA maturation before translation. The human genome contains ~10,000 *Alu* elements located in the 3′-untranslated region of coding genes, and 1% of them are active as polyadenylation sites (Chen et al., 2009). The vast majority of transcribed human pre-mRNA contains surprisingly high numbers of *Alu* elements, which likely have an essential role in adenosine-to-inosine (A-to-I) pre-mRNA editing (DeCerbo and Carmichael, 2005). Targets for editing are partially double-stranded RNA that is formed from the inverted repeats of conservative *Alu* sequences (IRAlu) localized in introns and untranslated regions, but not in coding regions. mRNAs without, or containing only low levels of inosine residues move into the cytoplasm and highly edited mRNA molecules are localized in the nucleus. As a result of editing, the expression of mRNA containing IR*Alu* can be modulated by regulating the quantity of mRNA arriving in the cytoplasm (Athanasiadis et al., 2004). Hyper-expression of *Alu* elements as a result of the suppression of the translation process by inhibitors (e.g., cycloheximide and pyromicin) reveals the close connection between the expression of *Alu* elements and the translation state within a cell (Liu et al., 1995). The expression of an *Alu* element is stimulated in response to various factors (e.g., viral infections, translation inhibitors, and factors of cellular stress) and it is believed they can participate in the regulation of translation during stress reactions (Rudin and Thompson, 2001). Up to 33% of all CpG sites in the human genome are located in *Alu* elements and their methylation is a primary mechanism of transposon activity suppression (Schmid, 1991; Slotkin and Martienssen, 2007). It is supposed that changes in the methylation status of *Alu* elements can act as global modifiers of gene expression and it is worth noting the inter-individual variability of the methylation profile of *Alu* elements; indeed, their epigenetic changes throughout life have been observed in monozygotic twins (Fraga et al., 2005; Sandovici et al., 2005). It was suggested that demethylation of *Alu* elements makes a significant contribution to global hypomethylation of the genome in aging (Bollati et al., 2009; Jintaridth and Mutirangura, 2010; Gentilini et al., 2012). Some research data reveal regulatory interactions between *Alu* elements and microRNAs (miRNAs) (Smalheiser and Torvik, 2006; Lehnert et al., 2009). The *Alu* elements localized in 3′-untranslated regions can serve as donors of miRNA target sites to various genes. Many of these genes are involved in regulation of transcription, cell cycle, cell proliferation, apoptosis, cell–cell contact, and signal transduction (Daskalova et al., 2006). miRNAs have recently emerged as important regulators of cellular senescence and aging (Smith-Vikos and Slack, 2012). As a result of genome-wide miRNA study, changes in miRNA expression with human aging were revealed (Elsharawy et al., 2012). Thus, it is clear the mobile elements connect different systems of regulation of gene expression, and it is important for understanding their role in aging.

The insertion/deletion polymorphism of *Alu* elements is widespread in human populations. Hypothetically, *Alu* element polymorphisms contribute to the variability of the human lifespan. Insertion of *Alu* elements into genes could cause epigenetic alterations and altered levels of gene expression, which is in accord with the results of some studies. For example, experiments with a cell line mono-allelic for *Alu* insertion/deletion have shown that the insertion of *Alu*Ya5 into the progesterone receptor gene *PGR* mediates the increased level of DNA methylation in the surrounding area of the genome, causes the inactivation of histone tail modifications and results in inactivation of the expression of neighboring genes (Byun et al., 2012).

In conclusion, *Alu* elements, which are components of the complex network of interrelated molecular and genetic changes; can have a role in structural and functional damage of the human genome during aging. Activation of *Alu* elements in response to environmental factors could be one of the triggers that mediate genome instability, alter the expression levels of genes, and lead to the gradual diminution of cell functions and organism functionality as a whole.
